# Kinematic and biomechanical estimates of head and neck loading during the long attack in IGP protection work dogs

**DOI:** 10.3389/fvets.2026.1778778

**Published:** 2026-04-15

**Authors:** Jose Manuel Vilar, Mirella Lopedote, Simona Valentini, Isabel Marrero, Giuseppe Spinella

**Affiliations:** 1 IUIBS, University of Las Palmas de Gran Canaria, Las Palmas de Gran Canaria, Spain; 2 Ambulatorio Veterinario Fisio & Sport, Trento, Italy; 3 Department of Veterinary Medical Sciences, University of Bologna, Ozzano dell’Emilia, Italy; 4 Hospital Clinico Veterinario, Universidad de Las Palmas de Gran Canaria, Trasmontaña, Spain

**Keywords:** Atlanto-occipital joint, biomechanics, high-impact activity, Internationale Gebrauchshund Pruefung dogs, kinematics, long attack

## Abstract

**Introduction:**

The long attack is a demanding exercise within the defence phase of IGP (Internationale Gebrauchshunde Prüfungsordnung) dog sport. The aim of this study was to describe the kinematics of the final approach and bite during the long attack and to estimate cervical mechanical loads acting on the atlanto-occipital (AO) joint using a simplified inverse dynamics approach.

**Methods:**

Ten well-trained Belgian Shepherd Malinois were evaluated. Kinematic variables, including velocity, flight time, and joint angles, were obtained from high-speed video recordings and analyzed using open-source motion analysis software. Mechanical variables (force, torque, power, and kinetic energy) were subsequently estimated from the kinematic data. Descriptive statistics were applied.

**Results:**

Mean (± SD) body kinetic energy at impact was 842.06 ± 243.03 J, and estimated torque at the AO joint was 333.72 ± 100.06 N·m. The total estimated force acting on the cranio-cervical region was 1439.29 ± 446.25 N.

**Discussion:**

These findings provide the first quantitative description of the mechanical demands imposed on the cranio-cervical region during the long attack. The estimated forces and torques at the AO joint suggest that this exercise generates substantial mechanical loading, highlighting the need for further research to explore potential long-term implications for cervical spine health in working dogs.

## Introduction

The Schutzhund discipline, formally known as IGP (Internationale Gebrauchshunde Prüfungsordnung), is a canine sport recognized by the FCI (1) and designed to evaluate working abilities in three phases: tracking, obedience, and defence (2, 3).

A specific component of the defence phase is the *long attack.* In the long attack the dog is placed in a basic seated position. The handler is allowed to hold the dog by the collar and the dog must remain calm and focused on the helper. The helper wears a protective paddle sleeve on one arm specifically designed to resist tearing and pressure from the dog’s teeth. When the helper has reached a distance of 50 meters from the handler and his dog, he assumes an attacking attitude; at the judge’s signal the handler releases the dog, which must react without hesitation to the simulated assault of the helper (1). After grabbing the sleeve, the dog is subjected to further pressure from the helper, who continues to swing a padded stick, without hitting the dog (“transition phase”) (1). When the transition phase is completed, the dog must release the sleeve on command (1).

The final phase of the long attack combines high acceleration with abrupt deceleration upon impact, resulting in a rapid transfer of kinetic energy that is largely absorbed by the cranio-cervical region (4, 5). This dynamic movement is biomechanically analogous to whiplash-like motion, as the head decelerates suddenly while the body’s momentum continues forward. Considerable stress is thereby imposed on the cervical spine, particularly on the atlanto-occipital (AO) joint, a key structure for force transmission between the head and trunk (4, 5).

The veterinary biomechanics literature has predominantly focused on activities such as agility, where the dog performs a jump with distinct take-off and landing phases, unlike IGP dogs (6–13). Despite the physical intensity of long attack and its possible repercussions on the dog’s body, scientific literature has focused mainly on bite force measurements (3, 14–16) while kinematic and inertial aspects of the approach and impact remain poorly characterized. In the authors’ opinion, this gap is particularly relevant for breeds commonly used in IGP and protection work, such as the Belgian Shepherd Malinois (BSM). The biomechanical demands of the *long attack* may vary depending on factors like body mass, approach velocity, jump distance, and individual bite strategy (17). Based on our experience, BSMs often exhibit a high-impact approach, sometimes overshooting the target, which may amplify mechanical load on cervical structures. Although some studies have examined canine acceleration in various contexts as long and hurdle jumps (17–20), deceleration forces during protection work—and their potential effect on cervical health—have not been adequately studied within IGP or police dog training.

The lack of detailed analysis of the *long attack*‘s final phase represents a significant research gap. Quantifying inertial forces and torque at the AO joint is a necessary step toward understanding the physical demands of this exercise. Beyond scientific interest, this knowledge may have implications for working dog well-being. It has been hypothesized that repetitive high-intensity actions, if not properly managed, could potentially lead to cumulative microtrauma or musculoskeletal injury, which might theoretically affect a dog’s functional career and quality of life (21–23). However, direct evidence linking these mechanical loads to specific injuries remains lacking.

The aim of this study was to perform a descriptive kinematic and mechanical analysis of the final bite phase during the long attack in BSMs, quantifying the mechanical stress experienced by the AO joint during this high-impact task.

## Materials and methods

### Study population

Ten adult, clinically sound BSMs of both sexes, regularly trained for IGP competitions, were included in the study on a volunteer basis. Further inclusion criteria required dogs to be declared physically fit for the study by their handlers following a veterinary evaluation. Additionally, dogs had to exhibit normal muscle mass (according to the Muscle Condition Score reported by Freeman et al. in 2019: normal muscle mass or mild, moderate, or severe muscle loss) (24) and be experienced in IGP competitions (IGP-3 level). Any evidence of lameness or macroscopic lesion (i.e., skin wound, joint effusion) determined the exclusion of the dog. Owner consent was obtained prior to participation and the study was approved by the Animal Welfare Committee of Bologna University (protocol number: ID 4491).

### Exercise design

**Figure 1:**
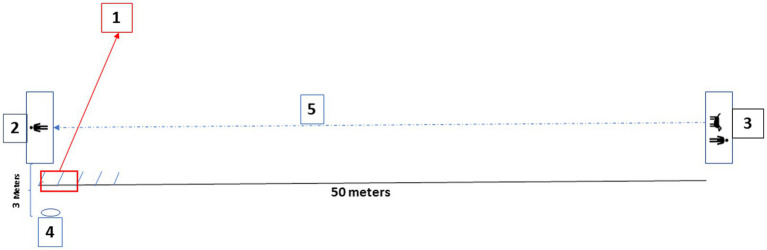
Schematic representation of the experimental setup showing: (1) The dog’s estimated jump trajectory; (2) the helper’s position within the reference rectangle; (3) the handler and dog starting position; (4) the tripod-mounted digital camera used for video recording, positioned at 3 m of distance from the dog’s estimated jump trajectory; (5) the trajectory followed by the dog from the starting point with the handler to the point of impact with the helper (total length of approximately 50 m).

All data were collected during a competition-level trial simulation in 1 day on outdoor flat grass surface during daylight hours under consistent lighting conditions to minimize variability. Dogs were warmed up starting with on-leash walking, gradually increasing to a trot for about 5 min prior to the trials and were directed by their handler throughout the entire study. The starting position of the dog complied with the international FCI rules; therefore, the dog was positioned 50 meters from the helper. The helper was already present in the area when the dog was brought into the field and he positioned himself within or as close as possible to a rectangular area measuring 1 × 2 meters, in order to minimize, as far as possible, non-calculable distance variables between the helper and the dog. After a specific order of the judge, the handler immediately released the dog with the command to go. The helper received the dog from the front, offering his sleeve. The dog had to bite the sleeve without slowing down before impact. After the grip the helper applied defensive pressure by wielding the padded stick, simulating continued agitation. At the order of the judge, the helper stopped the pressure and the dog had to release the sleeve. After the release, the dog had to remain close to the helper for approximately 5 s (1). Each dog performed at least one valid trial (i.e., complete attack as reported by FCI regulations) for analysis. Dogs were filmed during the final phase of the long attack. For data collection, a tripod mounted digital camera (iPhone 15 pro) 1.55 meters high from the ground was placed perpendicular to the canine left side at a distance of 3 meters away from the helper-dog contact point, and laterally, approximately at the midpoint of the dog’s estimated jump trajectory. Camera position and orientation were fixed across all trials and all dogs (Figure 1). The video acquisition was set at a resolution of 1080p and a frame rate of 120 fps.

### Kinematic analysis

**Figure 2:**
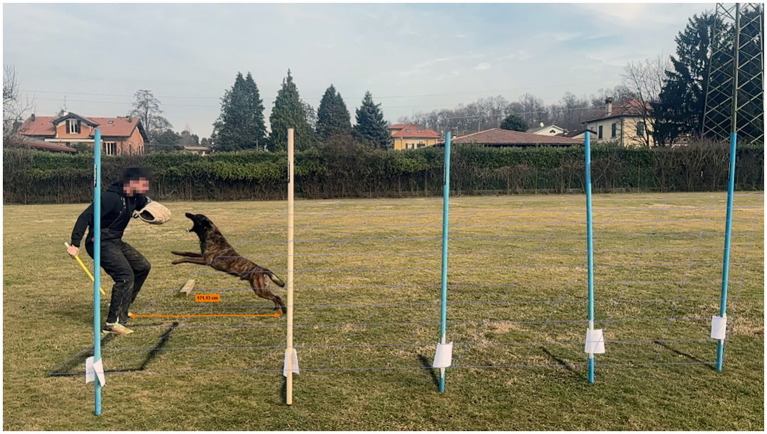
Measurement of jump distance. The helper remains positioned adjacent to the black reference rectangle (1 × 2 m). A calibrated perspective grid superimposed on the video footage was used to correct for parallax and enable distance quantification.

**Figure 3:**
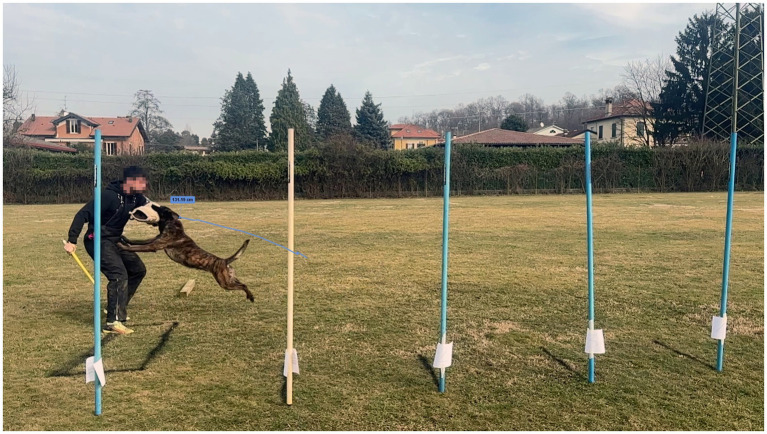
Measurement of flight length, defined as the distance along the jump trajectory from the moment of forepaw take-off to the moment of initial contact between the dog’s mouth and the helper’s sleeve.

**Figure 4:**
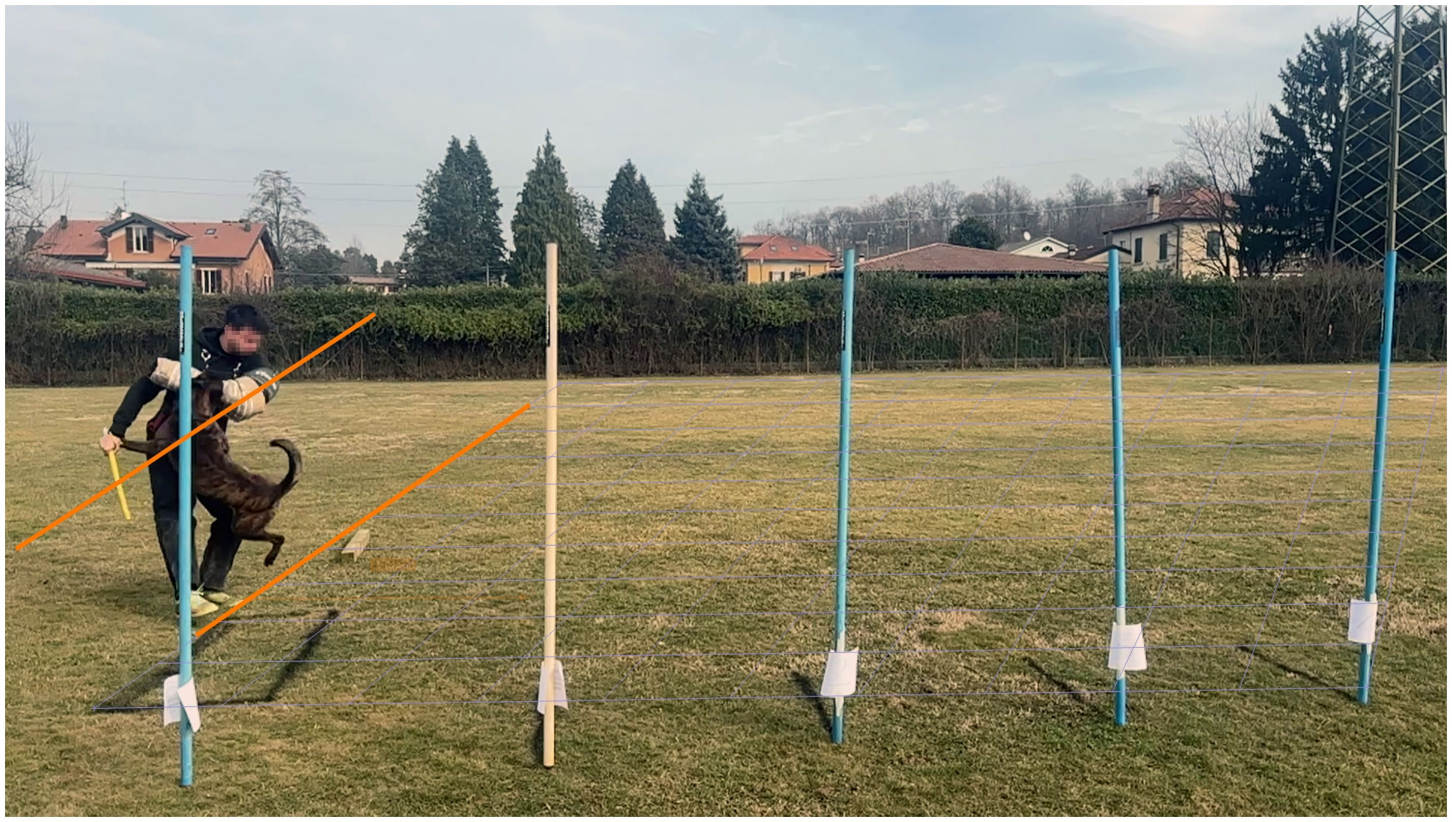
Determination of impact time. The impact phase was defined as the interval from first visible mouth-sleeve contact until the helper’s sleeve-holding arm became aligned with the dog’s transverse body plane (i.e., perpendicular to the direction of movement and parallel to the perspective grid).

**Figure 5:**
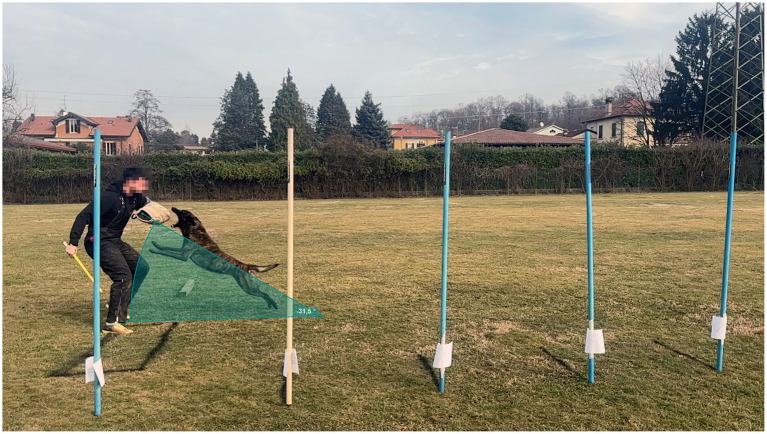
Measurement of body angle during the jump phase. Body angle was defined as the angle between the horizontal plane and the line connecting the dog’s ischiatic tuberosity and scapulohumeral joint.

**Figure 6:**
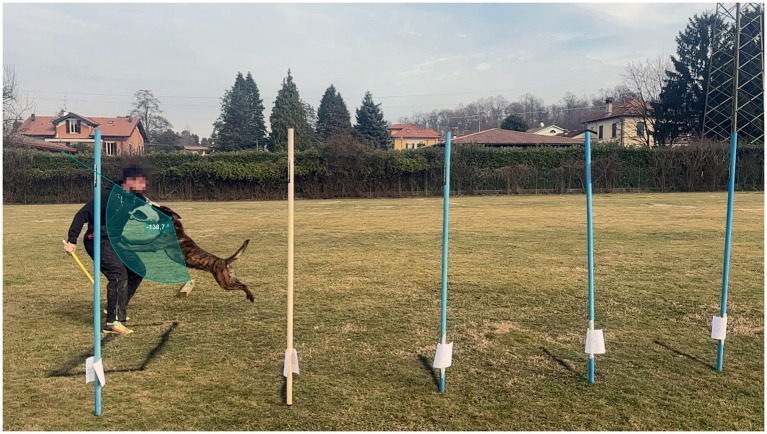
Measurement of head–neck angle, defined as the angle between the line connecting the external occipital protuberance to the nasal plane and the external occipital protuberance to the approximate position of the xiphoid process as the distal arm.

Video data were analyzed using Kinovea® (version 2024.1.1.), an open-source motion analysis software. No smoothing or filtering techniques were applied; all measurements were taken directly from raw frame-by-frame analysis. Specific anatomical landmarks were tracked to determine body displacement (caudal end of the withers), head trajectory (external occipital protuberance), and neck orientation (nasal plane, xiphoid process). All landmarks were identified and tracked frame by frame by a single experienced evaluator with advanced training in canine musculoskeletal anatomy and kinematic analysis. To minimize measurement error due to perspective distortion, a perspective grid tool was applied. This tool allowed calibration of the sagittal plane based on known physical dimensions in both the horizontal and depth axes, improving tracking accuracy for head and neck movement. For each valid dog trial, the following variables were measured:

- *Dog length* (cm): distance between the ischiatic tuberosity and the most cranial part of the scapulohumeral joint. A measurement tape was used for this purpose.
- *Jump distance* (cm): distance between the position of the helper feet and the dog hindlimbs when jump starts (take-off straight jump). If both hindlimbs were not at the same position at the initial phase of the jump (take-off wrap jump), mean distance between them was considered (Figure 2).
- *Flight time* (s): time in seconds from the forepaws take-off to the contact of the dog’s mouth with the helper’s sleeve.
- *Flight length* (cm): length of the flight trajectory from the moment that the front-paws leave the ground until the dog’s mouth contacts the helper’s sleeve. External occipital protuberance was taken as visual body reference (Figure 3).
- *Impact time* (s): time, measured in seconds, from the dog-sleeve contact to the instant in which the sleeve-arm reaches the transversal plane (Figure 4).
- *Body angle* (°): axial body angle in degrees with respect to the ground. The axial anatomical references were the ischiatic tuberosity and the scapulohumeral joint (Figure 5).
- *Terminal velocity* (m/s): defined as the maximum velocity reached by the dog’s body immediately before (8 ms or less) contacting the helper’s sleeve.
- *Head–neck angle* (° - degrees): angle in degrees between the dog’s head and neck. The anatomical landmarks were: the external occipital protuberance as the vertex, the nasal plane as the cranial arm, and a line extending from the external occipital protuberance to the approximate position of the xiphoid process as the distal arm (Figure 6).

Derived mechanical variables within the impact phase were calculated using standard biomechanical equations, including:

- *Angular velocity* (rad/s): referred to the head–neck rotational angle.
- *Torque* (N·m) at the AO joint. It was estimated using a simplified rotational model according to *τ* = I · *α*, where I is the moment of inertia of the head (kg·m^2^) and α is the angular acceleration (rad/s^2^).
- *Tangential equivalent force (TEF, N):* linear force that would produce the same rotational moment around the AO joint as the measured torque. TEF was calculated as TEF = τ / r, where r is the head–neck radius (m), defined as the distance from the estimated center of rotation at the AO joint to the center of mass of the head.
- *Kinetic energy* (KE, J): the energy of motion possessed by the dog. KE was calculated as KE = ½ · m · v^2^, where m is body mass (kg) and v is terminal velocity (m/s).
- *Impact power* (P, W): was defined as kinetic energy dissipated over the impact time: P = KE / t, where t is impact time (s).
-*Total power* (W/kg): power relative to body weight.
- *Terminal acceleration* (a, m/s^2^): defined as the estimated deceleration required to reduce the terminal velocity within the impact time: a = v/t.
- *G-force*: normalization to gravitational acceleration (G = a/g). Quantifies inertial loading during impact.

The designed model to obtain these parameters was simplified by considering that the head was considered as a rigid segment rotating around the AO joint, and motion was assumed to occur predominantly in the sagittal plane. Head mass was estimated as a fixed proportion of total body mass, and the head center of mass was assumed to lie along the head–neck axis.

Regarding the interpretation of torque and force values, reported torque values represent estimated joint moments acting at the AO joint. TEF did not represent a directly measured force but an equivalent tangential force applied at distance r that could produce the same joint moment. Linear force values represented estimated inertial forces associated with whole-body deceleration. Total estimated force reflected the combined rotational and linear components.

All biomechanical variables were subsequently calculated using custom spreadsheet-based calculations implemented in Microsoft Excel, following a predefined and consistent workflow linking kinematic inputs to derived mechanical outputs.

### Statistical analysis

All variability metrics (e.g., standard deviation, confidence intervals) reflect between-subject variability, as only one trial was analyzed per dog. Data were analyzed using Python 3.11 (SciPy and pandas libraries). For each variable, descriptive statistics were calculated, including mean, standard deviation (SD), and 95% confidence intervals (CI).

As this study was designed as a descriptive exploratory investigation, no *a priori* sample size calculation was performed. The sample size reflects the number of eligible, trained Belgian Shepherd dogs available during the study period.

## Results

Ten BSMs (4 intact females and 6 intact males) were included in this study population. The mean age (± standard deviation [SD]) of the dogs was 4.4 ± 1.9 (median 4 years) and mean (± SD) body weight was 29 ± 4.8 kg (median 28.5 kg). All 10 dogs fit the inclusion criteria.

The long attack sequence was divided into four key biomechanical phases: approach, take-off, flight, and impact. The following sections report the corresponding kinematic and mechanical variables.

Table 1 summarizes the morphological and flight-related kinematic parameters. Dogs showed a consistent body length (mean 61.4 ± 3.17 cm), with a mean jump distance of 171.86 ± 13.45 cm and flight time of 148.00 ± 28.46 ms. The body angle during flight averaged 28.44 ± 6.22 degrees, reflecting the trajectory adopted to reach the helper.

**Table 1:** Descriptive statistics for morphological and kinematic variables measured during the long attack in Belgian Shepherd Dogs (*N* = 10).

Variable	Mean ± SD	95% CI
Body length (cm)	61.40 ± 3.17	59.13–63.67
Jump distance (cm)	171.86 ± 13.45	162.24–181.49
Flight time (ms)	148.00 ± 28.46	127.64–168.36
Flight length (cm)	125.65 ± 26.61	106.61–144.68
Body angle (°)	28.44 ± 6.22	23.99–32.89

Mean values are presented with standard deviation (SD) and 95% confidence intervals (CI).

Terminal and energetic variables are presented in Table 2. The mean terminal velocity was 7.57 ± 1.01 m/s, with a mean kinetic energy of 842.06 ± 243.03 J at the moment of impact. The corresponding impact power was 14,514.38 ± 5,874.78 W. When normalized to body mass, relative power reached a mean value of 29.13 ± 8.52 W/kg. Terminal acceleration averaged 83.1 ± 14.44 m/s^2^, equivalent to a mean G-force of 8.47 ± 1.47.

**Table 2:** Descriptive statistics for terminal and energetic variables measured during the long attack in Belgian Shepherd Dogs (*N* = 10).

Variable	Mean ± SD	95% CI
Mass (kg)	29.00 ± 3.16	26.74–31.26
Terminal velocity (m/s)	7.57 ± 1.01	6.85–8.29
Impact time (s)	0.06 ± 0.01	0.06–0.07
Kinetic energy (J)	842.06 ± 243.03	668.21–1015.92
Impact power (W)	14514.38 ± 5874.78	10311.82–18716.94
Total power (W/kg)	29.13 ± 8.52	23.04–35.23
Terminal acceleration (m/s^2^)	83.10 ± 14.44	72.77–93.43
G-force	8.47 ± 1.47	7.42–9.52

Mean values are presented with standard deviation (SD) and 95% confidence intervals (CI).

Table 3 reports the variables related to cranio-cervical loading. The average head–neck angle was 162.26 ± 12.53 degrees. Mean angular velocity was 46.1 rad/s and the estimated torque at the AO joint was 333.72 N·m, and TEF was 1439.29 N.

**Table 3:** Descriptive statistics for mechanical variables measured during the long attack in Belgian Shepherd Dogs (*N* = 10).

Variable	Mean ± SD	95% CI
Head–neck angle (°)	162.26 ± 12.53	153.30–171.22
Angular velocity (rad/s)	46.10 ± 7.52	40.72–51.48
Torque (N·m)	333.72 ± 100.06	271.70–395.73
TEF (N)	1439.29 ± 446.25	1162.24–1715.94

Mean values are presented with standard deviation (SD) and 95% confidence intervals (CI). TEF, Tangential Equivalent Force.

## Discussion

This study provides the first detailed description of the kinematic and mechanical demands of the long attack exercise in IGP-trained BSMs.

Regarding kinematic findings, this specific high-impact exercise combines substantial horizontal acceleration with an abrupt deceleration upon impact, generating considerable inertial forces acting on the cranio-cervical region. Biomechanically, this sequence presents kinematic similarities with the dynamics of a rear-end vehicle collision, where the head and neck experience a whiplash-like motion due to the abrupt transfer of momentum (4, 5). This analogy serves as a conceptual framework for understanding the inertial demands imposed on the cranio-cervical region, rather than implying validated tissue-level loading. However, to our knowledge, no previous studies have reported AO mechanical loads during working dog bite exercises. Thus, any extrapolation to injury risk has to be interpreted with caution.

The use of external anatomical landmarks to estimate joint kinematics has been previously reported in multiple canine biomechanics studies (7, 12, 18–20). In our investigation, the AO joint was selected as the primary reference point due to its anatomical role as the interface between the skull and cervical spine, and its mechanical relevance during deceleration in the bite phase. Although our approach cannot capture true intervertebral motion, we estimated the angular relationship between head and neck using consistent surface markers, and we modeled the joint as a hinge to approximate torque and rotational forces. This simplified method provides a repeatable and functionally meaningful estimation of cervical loading in working dogs performing high-impact tasks.

Regarding mechanical estimates on AO joint, the estimated torque force and peak linear forces suggest that this joint receives significant stress, which could theoretically contribute to cervical overload over time. Beyond the traditional focus on bite force (3), these results highlight the need to examine the kinetic and inertial aspects of protection work, particularly during high-speed impact such as the long attack, and to adopt training methods that strengthen the neck and head muscles (25, 26).

The descriptive data obtained in this study reveal that BSMs develop a dynamic strategy where horizontal propulsion predominates, optimizing speed and maintaining a proper elevation to approach the sleeve. The mean terminal velocity (7.57 ± 1.01 m/s) further supports the hypothesis that BSMs prioritize momentum and speed over precise trajectory control, a pattern consistent with their reputation for high-drive and forceful impact. A significant methodological challenge arises when trying to determine the exact duration of the impact phase during the long attack exercise. The interaction between the dog and the helper requires both parties to make complex dynamic adjustments, unlike controlled mechanical systems. Upon contact, the helper instinctively flexes the arm and rotates the torso laterally to absorb the incoming force and minimize injury. The variability introduced by this behavior makes it challenging to establish a universally valid endpoint for the impact (27).

Several alternative criteria could be proposed to delimit this phase, such as the moment when the elastic sleeve compresses maximally (3), or when the dog’s hindlimbs make contact with the ground. However, these events occur with inconsistent timing across individuals and may not always reflect the actual mechanical endpoint of energy transfer. For example, some dogs land simultaneously with the bite, while others remain airborne slightly longer, depending on their jump trajectory and angle of attack.

In this study, the impact time was based on video frame analysis as the interval between the first visible contact of the dog’s mouth with the sleeve and the instant when the helper’s arm aligns with the torso in the transverse plane during the rotation maneuver. While this criterion is not perfectly objective, it provides a relatively consistent anatomical reference point across trials and individuals. To our knowledge, there is no recognized standard for determining impact time in canine bite work, and the chosen approach provides a pragmatic balance between repeatability and biomechanical importance. When comparing working trial context to agility, these distances and angles differ substantially. Miró et al. reported mean take-off angles of 42°–48° in agility hurdle clearance, which reflects a much steeper trajectory to negotiate vertical obstacles. The same authors observed that dogs who perform long jumps in agility usually maintain lower horizontal speeds (typically 4–5 m/s) and exhibit longer aerial phases compared to the rapid and flat trajectory of the *long attack* (19). Williams et al. and Carter et al. documented that forelimb joint kinematics during agility jumps involve progressive deceleration before landing, a strategy absent in the *long attack* where the landing surface is not the ground but an actively resisted sleeve (17, 20). These comparisons highlight the unique biomechanical profile of the *long attack*: an explosive, high-speed movement terminating in a collision rather than an absorptive landing.

Also, literature directly related to deceleration forces during protection work is extremely limited. Hyytiäinen et al. reported peak accelerations of up to 11.6 g in modified long attack exercises, values consistent with those observed in our study and indicative of the intense mechanical loading at the moment of impact (3). Indeed, specific studies regarding biomechanics of the canine cervical spine indicate that even moderate cervical muscle activation may generate substantial joint moments at the AO and atlanto-axial joints (28). Evidence from human biomechanics offers a useful comparative framework, as whiplash injury has been described biomechanically as a rapid acceleration–deceleration event involving an initial flexion deformation, followed by an S-shaped curvature and a final hyperextension phase, during which high strains are imposed on the facet joint capsules, structures commonly associated with cervical pain (4). While this comparison provides a conceptual basis for interpreting the inertial patterns observed in dogs, direct extrapolation of tissue-level loading between species is not possible. Despite this, the high kinetic energy and abrupt deceleration observed during the long attack suggest that this exercise may impose cervical loading patterns comparable to those reported in both canine and human models, supporting the need for further investigation into whether repeated exposure to such loading patterns may have clinical implications for IGP dogs. In this context, while bite force studies have traditionally dominated the research landscape, no research studies addressing the combined effect of translational and rotational forces on the cervical spine during these exercises have been found.

In contrast, agility research provides indirect insights into the consequences of repetitive high-impact actions. Williams et al. demonstrated that changes in jump height or distance significantly influence joint loading (17), and Carter et al. linked repetitive hurdle clearance to increased forelimb peak vertical forces, suggesting that cumulative exposure to extreme kinematic demands could predispose to musculoskeletal injury (20, 29). Essner et al. further reported a high prevalence of thoracic, lumbar and lumbosacral spine injuries in Swedish working and sport dogs, reinforcing the importance of investigating mechanical stressors beyond conventional ground impact (30). Interestingly, this study shows that neck injuries are not particularly common in bite work class. These findings collectively emphasize that while the *long attack* is unique in its kinematic and inertial characteristics, its potentially harmful profile may share parallels with other high-intensity canine sports.

The kinematic and mechanical parameters characterized here serve as a basis for optimizing performance and, potentially, preventing injury in working dogs. The combination of horizontal momentum and abrupt deceleration creates a scenario where the AO joint must absorb substantial torque while stabilizing the head. Given its anatomical function, this joint may be susceptible to significant mechanical stress in this setting (31–33).

If repeatedly experienced, such loading could theoretically contribute to soft tissue strain—highlighting a potential role for conditioning. Based on observational experience, BSMs may benefit from individualized training protocols and progressive loading strategies to mitigate this hypothetical risk. Recent studies indicate that the ability to counteract forces may be as relevant as the forces themselves (34, 35). Thus, it is prudent to consider conditioning programs aimed at improving core and cervical muscle stability, with the hypothesized objective of mitigating injury risk and extending career longevity (25, 26), although this hypothesis warrants further empirical validation. From a well-being perspective, understanding these loads may contribute to preserving functional longevity in working dogs.

Injury prevention not only reduces the physical and emotional burden on the dog, but also maintains operational reliability, a priority for working dog programs worldwide (30).

This study has several limitations. The sample size was limited, and mechanical estimations relied on video-based kinematic analysis rather than direct force plate measurements or *in vivo* sensor recordings. While validated biomechanical equations were applied, simplifications were necessary for modeling head–neck dynamics. Additionally, the absence of clinical outcome data precludes any definitive association between mechanical loading and injury occurrence. On the other hand, this study relied on a single-camera, two-dimensional video analysis using Kinovea, a methodology that carries inherent limitations. As highlighted by Winkler et al. (36), 2D motion analysis is susceptible to projection errors, particularly when out-of-plane movement occurs (36, 37). While the sagittal camera orientation in our study was chosen to align closely with the expected trajectory of the dog’s movement, some residual parallax or off-plane motion may have influenced angular and positional estimates. Furthermore, frame rate and manual tracking accuracy can significantly affect the reliability of peak values and derived variables, such as angular velocity or impact acceleration. Additionally, the estimation of the AO joint position based on surface anatomical landmarks introduces an element of uncertainty, as joint centers are not directly observable and must be inferred. Although we attempted to minimize these effects through standardized procedures and consistent landmark identification, we acknowledge these methodological constraints and their potential influence on the precision of the biomechanical estimates presented.

Ultimately, the biomechanical calculations presented in this study are model-dependent estimates derived from a simplified rigid-body representation of the head–neck complex. Assumptions regarding head mass fraction, center of mass location, lever arm radius, and the inferred position of the AO joint directly influence the resulting torque and force magnitudes. Small deviations in these assumed segment parameters may substantially affect the absolute numerical values reported. Therefore, the presented mechanical variables should be interpreted as approximations of relative loading conditions rather than exact *in vivo* joint forces. While this simplified modeling approach allows practical estimation under field conditions, it inherently limits the precision of absolute magnitude interpretation.

Future studies should incorporate wearable inertial sensors to capture real-time acceleration and force data, and use pressure-sensing collars to validate torque estimations at the AO joint. Comparative analyses across breeds, training styles, and experience levels and potential influence of the helper movement will help clarify whether biomechanical loading patterns differ substantially between dog populations. Finally, coupling biomechanical assessment with prospective injury surveillance could establish clinically relevant thresholds for safe training and competition in protection sports.

## Conclusion

This study provides the first detailed, descriptive kinematic analysis of the long attack in IGP-trained Belgian Shepherd Malinois. Our findings quantitatively characterize this specific exercise as a high-speed, high-impact maneuver that generates substantial linear and rotational accelerations at the cranio-cervical region, with mean impact forces and torques at the AO joint reaching considerable magnitudes. The movement pattern, characterized by horizontal propulsion and abrupt deceleration upon collision, presents a unique biomechanical profile distinct from other canine activities like agility jumping. While our simplified, model-based estimations are subject to limitations inherent to 2D kinematic analysis and the absence of *in vivo* validation, they offer a foundational reference for the mechanical demands of this task. These descriptive data suggest that repetitive exposure to such loads may contribute to cervical spine overuse injuries. However, due to the absence of clinical outcome data and the exploratory nature of our models, any inference regarding injury risk remains speculative. Future research should prioritize longitudinal studies combining biomechanical assessment with injury surveillance to explore this hypothesis and establish evidence-based guidelines for training and conditioning in working dogs.

## Data Availability

The raw data supporting the conclusions of this article will be made available by the authors, without undue reservation.
